# Effect of Thermal Treatment on Fracture Properties and Adsorption Properties of Spruce Wood

**DOI:** 10.3390/ma6094186

**Published:** 2013-09-18

**Authors:** Koji Murata, Yasuhiro Watanabe, Takato Nakano

**Affiliations:** 1 Division of Forest and Biomaterials Science, Graduate School of Agriculture, Kyoto University, Kitashirakawa Oiwake-cho, Sakyo-ku, Kyoto 606-8502, Japan; E-Mail: tnakano@kais.kyoto-u.ac.jp; 2 Canon Inc., Ota-ku, Tokyo 146-8501, Japan; E-Mail: watanabe.yasuhiro548@canon.co.jp

**Keywords:** wood, thermal treatment, fracture energy, strain-softening behavior, fiber saturation point, adsorption isotherm

## Abstract

The effect of thermal treatment on spruce is examined by analyzing the fracture and hygroscopic properties. Specimens were heated at temperatures within the range 120–200 °C for 1 h. Fracture energy was measured using a single-edge notched bending test and the strain-softening index was estimated by dividing the fracture energy by the maximum load. Adsorption properties were estimated using adsorption isotherms. Fiber saturation points (FSPs) were estimated by extrapolating the moisture adsorption isotherm curve. Langmuir’s adsorption coefficient and number of adsorption sites were obtained using Langmuir’s theory and the Hailwood-Horrobin theory, respectively. The fracture energy, FSPs, and specimen weights decreased at temperatures higher than 150 °C, but the critical point for the strain-softening index and the number of adsorption sites was shown to be 180 °C. We hypothesize that the fracture energy and FSP depend on the chemical structure of the cell wall, whereas the strain-softening behavior may be influenced by the number of adsorption sites, and in turn the number of hydrogen bonds in hemicellulose.

## 1. Introduction

Research has long been conducted on controlling the mechanical and adsorption properties of wood by thermal treatment. The hygroscopic properties of wood are attenuated by heating at high temperatures [1,2,3]. However, it has been reported that the mechanical properties of wood are degraded on heating [4]. The adsorption properties are thought to depend on the chemical structure of the wood substance [3], as well as on the ultrastructure of the cell wall. Similarly, the strength and other mechanical properties of wood are affected by anatomical properties, cell wall ultrastructure and chemical composition [5]. Therefore, thermal treatment to change the mechanical properties of wood would lead to similar changes in the adsorption properties; however, to date there has been no study reporting the effects of thermal treatment on both adsorption and fracture properties. Hill *et al.* [6] described the sorption behavior of wood using the parallel exponential kinetics (PEK) model with the modulus of elasticity and viscosity of the cell wall. Here, we focus on the fracture behavior of wood. The current study has addressed this question in an attempt to provide new knowledge about wood structures and chemical substances.

The aim of this study is to elucidate the effect of thermal treatment on the chemical structure of the cell wall and the fracture properties of wood. The chemical structure of wood cell wall is quantified by estimating adsorption properties based on characterization theories, Langmuir’s theory [7], and Hailwood–Horrobin theory [8]. The number of adsorption sites and the mechanical strength of veneer sheets of poplar wood were decreased by heating at temperatures higher than 180 °C [9]. Jakob *et al.* [10] investigated the structural changes in natural *Picea abies* wood cells by examining the moisture content at the nanoscale level using small-angle X-ray scattering (SAXS). It was found that considerable changes in the structure of cell wall occurred in specimens dried at the temperature below the fiber saturation point (FSP). At moisture contents above FSP, however, the ultrastructure of the cell wall was independent of hydration; FSP may depend on the ultrastructure of cell wall. In this study, FSP is estimated by extrapolating the adsorption isotherm of the heated wood specimens and compared to their mechanical properties. Hill [11] said that the extrapolation method using sorption isotherms do not yield the same properties as saturation methods such as solute exclusion or pressure plate methods. Although the FSP determined by adsorption isotherms is not equal to true FPS, the FSP is thought to depend on the chemical properties of wood. In this study, therefore, we examined the FSP from adsorption isotherms in order to discuss the thermal treatment. Fracture properties of wood specimens, such as fracture energy and maximum load, are obtained by a single-edge notched bending (SENB) test [12].

## 2. Materials and Methods

### 2.1. Thermal Treatment

Air dried spruce wood (*Picea* sp.) specimens with a dimension of 40 mm × 40 mm × 15 mm were cut from two boards. These specimens were dried in a conditioning room maintained at 20 °C and 65% relative humidity (RH) for a few weeks and in a vacuum chamber at 60 °C for one day. The specimens dried in a vacuum chamber without additional heating are denoted as non-heated specimens. The remaining specimens were thermally treated for 1 h at increments of 10 °C between 120 and 200 °C in an oven drier (DX602, YAMATO, Tokyo, Japan).

### 2.2. Fracture Test

**Figure 1:**
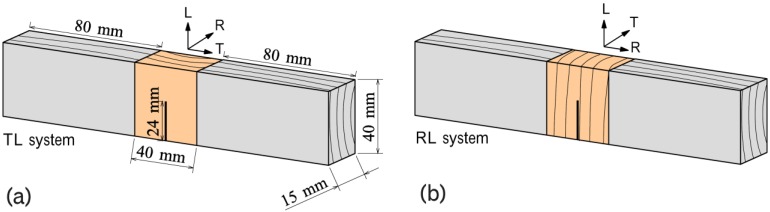
Specimens used in the single-edge notched bending test; (**a**) TL system (Tangential-Longitudinal); (**b**) RL system (Radial-Longitudinal).

The fracture properties of heated specimens were measured using an single-edge notched bend (SENB) test. Two types of specimens were prepared for the fracture test (Figure 1). For each thermal treatment, eight specimens, with an average density of 390 kg/m^3^, were tested in a TL system (Tangential-Longitudinal), where the first character indicates the direction normal to failure plane and the second character indicates the direction in crack progress. Five specimens, with an average density of 470 kg/m^3^, were tested in a RL system (Radial-Longitudinal). Since TL system specimens were cut from a single board and RL system specimens were from another board, the average densities were not equal. Two spruce support blocks (80 mm × 40 mm × 15 mm) were glued to the heated specimens. The starter notch of 24 mm was cut along the fiber direction using a small band saw (Figure 1). The final cut of 1 mm on the top of the starter notch was made using a razor blade. The SENB specimens were kept in a conditioning room (20 °C and 65% RH) for two weeks. The SENB test was performed using a material testing machine (AG-I/100kN, Shimadzu, Kyoto, Japan), which measured the load and displacement at the center point. The rate of the crosshead movement was 1 mm/min, and the span of the supports was 160 mm. The fracture energy *G* was calculated by substituting the values of load *P* and displacement δ in the following equation:

(1) $$G=\frac{mg\delta_{0}+\int_{0}^{\delta_{0}}Pd\delta }{A}$$

where *A* is the cross-sectional area of the ligament, *m* is the weight of the specimen, δ_0_ is the deflection when the specimen falls, and *g* is the acceleration due to gravity.

### 2.2. Moisture Adsorption Isotherm

The heated specimens were cut into smaller pieces of 5 (Longitudinal) mm × 15 (Radial) mm × 38 (Tangential) mm. The small specimens were dried in a vacuum chamber at 60 °C for one day and then conditioned for one month in a chamber containing prescribed saturated solutions maintained at 23 °C for obtaining an appropriate moisture content. The solutions used were LiCl (11% RH); CH_3_COOK (22% RH); MgCl_2_ (33% RH); K_2_CO_3_ (43% RH); LiNO_3_ (47% RH); Mg(NO_3_)_2_ (53% RH); NaBr (57% RH); NH_4_NO_3_ (62% RH); SrCl_3_ (71% RH); NaCl (75% RH); (NH_4_)_2_SO_4_ (80% RH); KCl (84% RH); BaCl_2_ (88% RH); KNO_3_ (92%RH); Pb(NO_3_)_2_ (95% RH); and K_2_SO_4_ (97% RH). Adsorption isotherm curves were obtained from the equilibrium moisture content (EMC) values of the heated specimens by fitting with a cubic polynomial equation.

Moisture adsorption properties were estimated on the basis of characterization theories. At first, the modified adsorption coefficients, *b*′, were obtained by fitting Langmuir’s adsorption equation [Equation (2)] [9,13] to EMC at 11%, 22%, 33%, and 44% RH.

(2) $$\frac{h}{m}=\frac{1}{b^{\prime }m_{0}}+\frac{h}{m_{0}}$$

In Equation (2), *m* and *m*_0_ represent the moisture content (g/g) and saturation concentration (g/g), respectively, and *h* represents the fractional RH. Next, the number of adsorption sites was estimated using the Hailwood–Horrobin theory [3,9]. Because the Hailwood-Horrobin adsorption equation [(Equation (3)] can be adopted for all humidity values, the absorption parameters were determined with EMCs at all of sixteen RH values tested.

(3) $$\frac{h}{m}=-ah^{2}+bh+c$$

(4) $$W=18\sqrt{b^{2}+4ac}$$

where *W* indicates the mass of adsorptive substance per mole of adsorption site. The number of adsorption sites is the reciprocal of *W* (mol/g). Last, the FSP of the heated specimen was obtained by extrapolating the adsorption isotherm curve at the saturated condition of *h* = 0.995 [14].

## 3. Results and Discussion

### 3.1. Fracture Properties

#### 3.1.1. Fracture Energy and Weight Loss on Thermal Treatment

**Figure 2:**
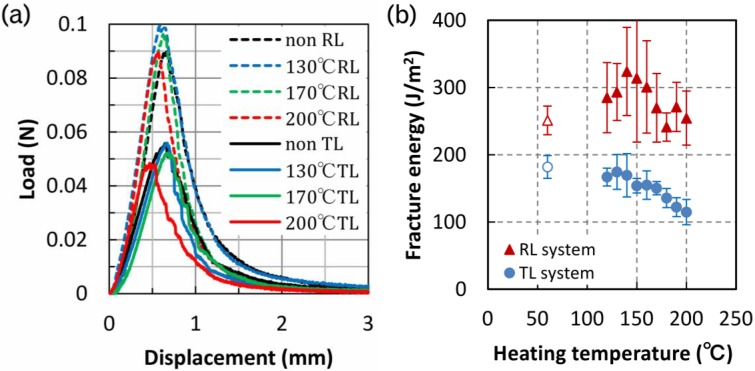
Changes in fracture energy: (**a**) load–displacement curves in single-end notched bending test and (**b**) fracture energy dependence on heating temperature.

Figure 2a shows the representative load–displacement curves obtained from the SENB test, with the obtained fracture energy shown in Table 1. The density of the TL system specimens was larger than that of the RL system specimens because the specimens were cut from two boards. Thus, it is difficult to compare the values of each property. We can, however, consider the temperature, which in turn affect the properties. Figure 2b shows the change in fracture energy with increasing temperature. At temperatures above 140 °C, the fracture energy decreased linearly in the TL system. In the RL system, the peak appeared around 150 °C, but the fracture energy measurements varied widely. The fracture energies of the RL specimens were higher than those of the TL specimens. In the fracture plane of the TL specimens, the cells are regularly arranged in straight lines in the radial direction, whereas in that of RL specimens, cells are arranged irregularly in the tangential direction. The fracture energy is thought to be related to the arrangement of the cell. Keuneche *et al.* [15] observed crack propagation both in the radial-tangential plane and in the tangential-radial plane in a micro wedge splitting test. In the TR system specimen, the crack path typically followed interfaces between rays and tracheids, which are well known as planes of weakness. On the contrary, in the RT system specimen the crack path was not straight. In the current study, the crack paths are thought to be similar to results reported by Keuneche *et al*. [15]. The fracture energy may depend on the arrangement of the cell and the actual area. The crack path in the TL system exhibits mainly intercellular fracture (separation of cells along their middle lamella), and that in the RL system displays both intercellular and transwall fracture.

**Table 1:** Fracture energy (in J/m^2^) of heated specimen in a single-end notched bending text.

Specimen	Density *	Non-heated	130 °C	150 °C	170 °C	200 °C
RL system	390 kg/m^3^	251	293	214	270	255
TL system	470 kg/m^3^	182	175	154	150	115

Note: * The densities of the specimen before thermal treatment.

**Figure 3:**
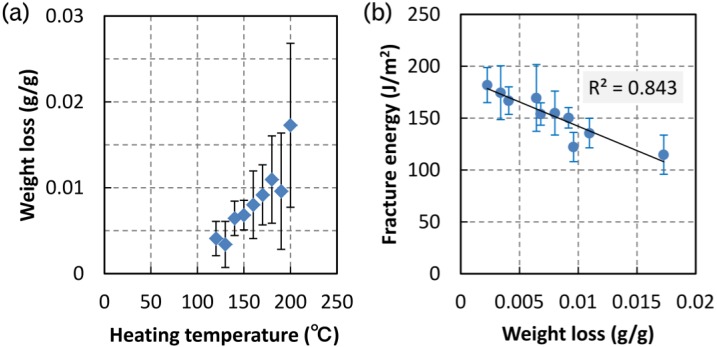
Effect of weight loss on fracture energy in tangentially loaded specimens; (**a**) weight loss by heat treatment and (**b**) fracture energy dependence on weight loss.

The weight loss of the TL specimens is shown in Figure 3a. Eight specimens were tested at each heating temperature between 120 and 200 °C. Error bars indicate the standard deviation. The weight loss began to decrease at 140 °C, after which it increased with the heating temperature, which agrees with the results of previous studies on the weight loss of poplar wood undergoing thermal treatment [9]. Obataya *et al.* [16] reported that the weight loss for spruce specimens was 0–0.01 (g/g) after heating at 120–180 °C for 1 h. The weight loss obtained in this study agreed with these reported data. Figure 3b shows the relationship between the fracture energy and the weight loss of TL specimens. The fracture energy decreased linearly with increasing weight loss, indicating that the thermal decomposition of wood affects the fracture energy. Thus, the micro- and nanostructure within the cell wall may be changed by thermal treatment.

#### 3.1.2. Maximum Load and Strain-Softening Index

**Figure 4:**
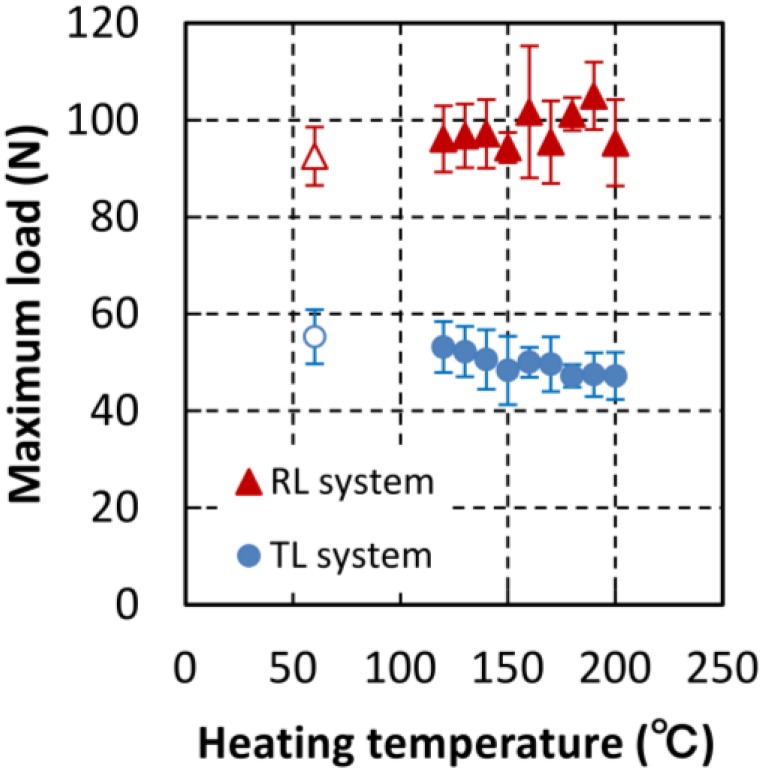
Dependence of maximum load on heating temperature.

Figure 4 shows the maximum loads in tests of the TL and in RL systems, where error bars indicate the standard deviation. Similar to the fracture energy, the maximum load of RL specimens was significantly larger than that of the TL specimens. In the RL system, the maximum load slowly increased with heating temperature, and varied irregularly at temperature above 150 °C. On the contrary, in TL system tests, the maximum load slowly decreased with heating temperature and saturated at temperatures above 150 °C. It is postulated that the maximum load depends on the conditions at the top of the starter notch and not on the crack path. The maximum load may be influenced by the weakest part of the anatomical structure of the wood at the top of the starter notch.

**Figure 5:**
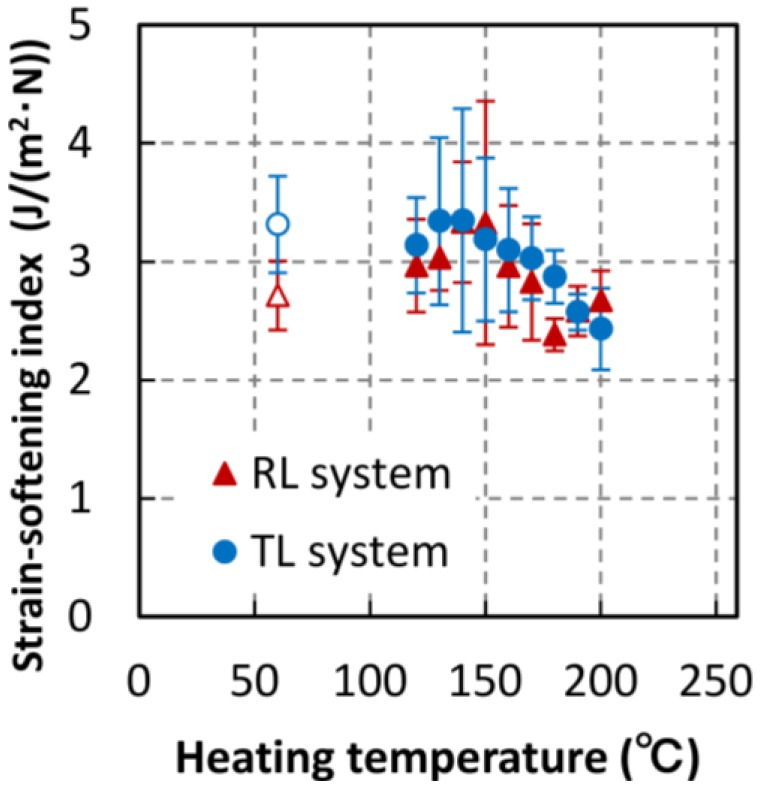
Strain-softening index obtained by dividing the fracture energy by the maximum load.

In this study, the strain-softening index is defined as the relative value obtained by dividing the fracture energy by the maximum load. The relationship between the fracture energy and the heating temperature was slightly different from that between the maximum load and the heating temperature. In order to estimate the quasi-brittle behavior [17], the strain–softening behavior after peak load was considered. Because the effect of maximum load on fracture energy was excluded, the relative fracture energy was thought to show the strain-softening behavior. Figure 5 shows the relationship between the strain-softening index and heating temperature, where error bars indicate the standard deviation. The strain-softening index slightly decreased at temperatures above 140 °C, and the trend changed in the range 180–190 °C. The variance of the data decreased with increasing temperature and reached its minimum value at 180–190 °C. We hypothesized that properties of or related to the toughening mechanism may have been altered by thermal treatment above 180 °C. From Figure 2, Figure 4 and Figure 5, we can determine that the mechanical properties of the RL specimens varied more widely and irregularly than those of the TL specimens. Taking the small number of specimens into consideration, only data collected from the TL systems will be shown hereafter because data collected from the RL systems were inconclusive.

### 3.2. Adsorption Properties

**Figure 6:**
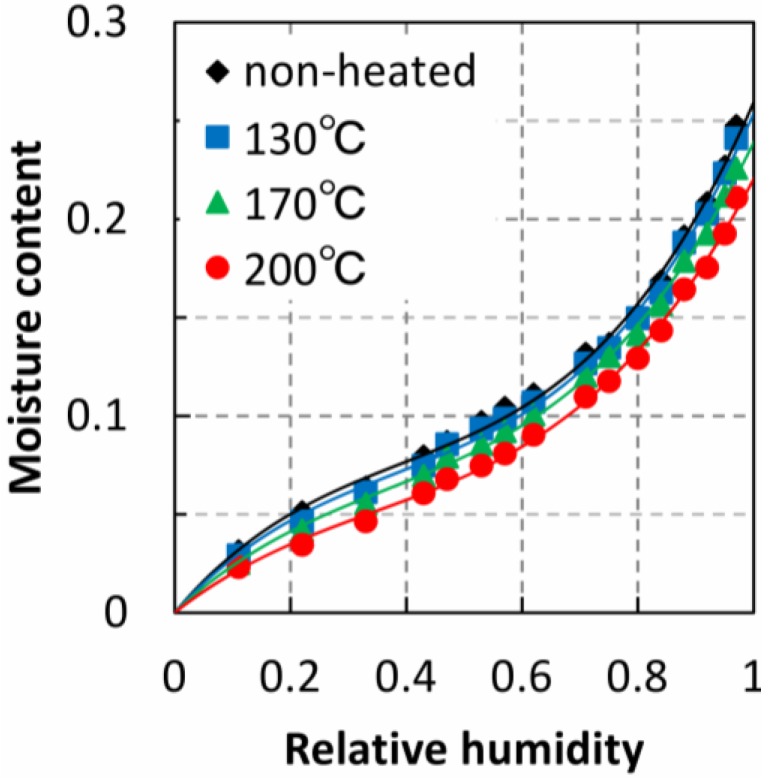
Moisture adsorption isotherm curves of heated specimens.

The adsorption isotherms obtained by EMC measurements are shown in Figure 6. Ten isotherm curves were obtained in total, and four representative curves were chosen for example. The EMC of the heated specimens decreased with increasing temperature, and the adsorption isotherm curves showed lower moisture content. Three adsorption properties were determined by the adsorption isotherms: the FSP, Langmuir’s modified adsorption coefficient, and the number of adsorption sites. Correlation coefficients for the relationship between the adsorption properties and the fracture properties are shown in Table 2. We found that the adsorption properties have a strong relationship with the fracture properties. The coefficients of determination indicating the strongest relationship are shown with underscore.

**Table 2:** Coefficients of determination* calculated for linear relationships between adsorption properties and fracture properties in the TL system.

Adsorption property	Fracture energy	Maximum load	Strain-softening index
Fiber saturation point	0.943	0.670	0.914
Modified adsorption coefficient	0.718	0.769	0.542
Number of adsorption site	0.855	0.834	0.885

Note: * The Pearson product-moment correlation coefficient.

#### 3.2.1. Fiber Saturation Point

**Figure 7:**
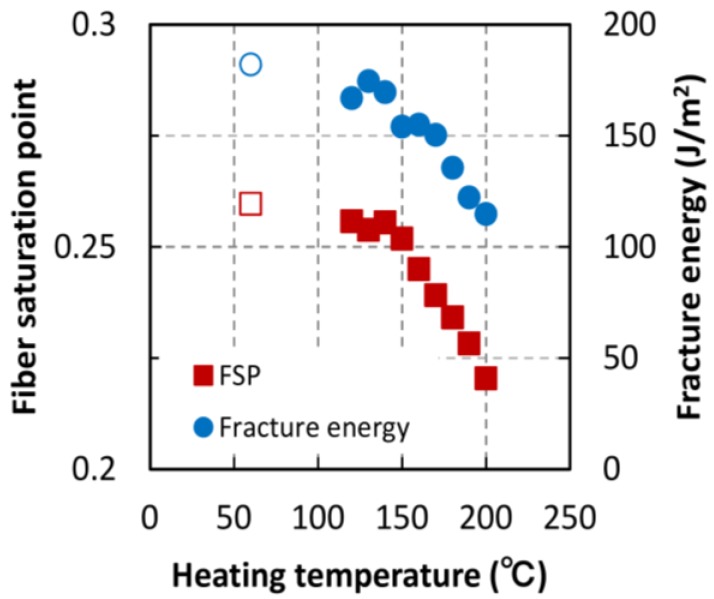
Fiber saturation point values obtained using adsorption isotherm curves. Open symbols indicate the data without heat treatment.

The FSP values of the TL specimens estimated using adsorption isotherms are shown in Figure 7, where the open marks indicate the FSP of the non-heated specimen. FSPs began to decrease at heating temperature of 150 °C, and continued decreasing linearly with temperatures above 150 °C. The fracture energies of the TL specimens are also shown in Figure 7. The decrease in fracture energies was similar to the decrease in FPSs and both properties exhibited this decrease at 150 °C. It is thought that the main factor affecting the FSP is the decrease of adsorption sites (*i.e.*, number of hydroxyl groups) caused by heating. However, this decrease could not be found until the heating temperature 170 °C in previous reports [9]. Rautkari *et al.* [18] said that a generally poor correlation between the EMC and hydroxyl group accessibility was found, and therefore additional mechanism exercises control over the EMC. Another factor is thought to be the restriction of the cell wall ultra-structure, so-called cross-band effect of the S1 layer [19]. The restriction is due to microfibrils in the S1 and S2 layers in the wood cell wall. FSPs obtained using the isotherms depended on EMC values at a high RH. Hill *et al.* [6] analyzed the sorption behaviors using the PEK model, and found that thermal modification increased the slow process viscosity which was an observation consistent with the known effects associated with destruction of the hemicellulose. The adsorption isotherm may be also affected with the mechanical deformation depending on elasticity and viscosity of the cell wall. Using small-angle X-ray diffraction, it was previously confirmed that the ultrastructure of the cell wall changed at high moisture contents [10]. Our results indicate that the ultra-structure of the cell wall begins to change at 150 °C and that this has an effect on the FSP. The change of the ultra-structure also decreased the mechanical properties of the cell wall. Both the FSP and the fracture energy are thought to depend on elasticity and viscosity of the cell wall.

#### 3.2.2. Langmuir’s Modified Adsorption Coefficient

**Figure 8:**
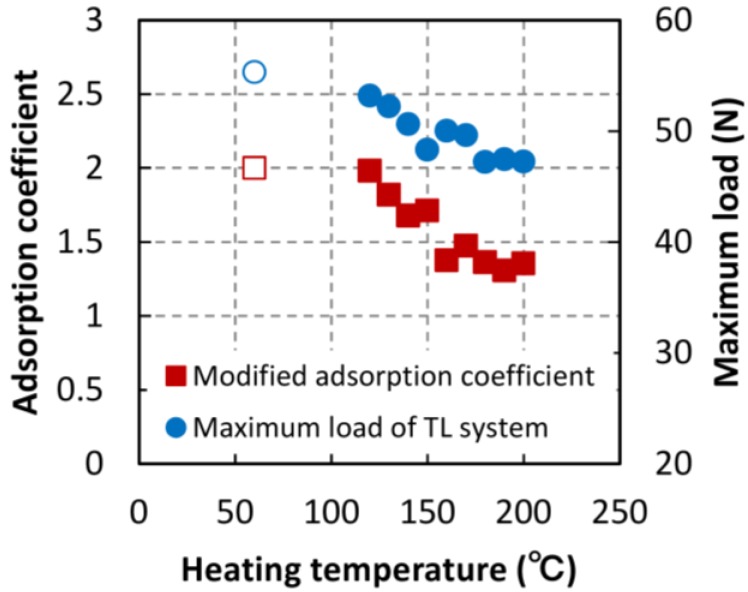
Langmuir’s modified adsorption coefficients for heated specimens.

Moisture adsorption isotherms at a lower RH were fitted by Equation (2) and Langmuir’s modified adsorption coefficients obtained from the fit are shown in Figure 8. The adsorption coefficients began to decrease with increasing heating temperature and saturated above 150 °C. Langmuir’s modified adsorption coefficient is exponentially related to the positive value of the adsorption energy, *E*, as shown in Equation (5) [20]:

(5) $$b^{\prime }=bp_{0}=p_{0}K\exp(\frac{E}{RT})$$

where *R* is the universal gas constant (8.314 J/mol∙K), *T* is the temperature (K), *p* is the vapor pressure and *p*_0_ is the saturated vapor pressure (23.38 hPa at 20 °C). The pre-exponential factor, *K*, is equal to the ratio of the adsorption and desorption coefficients. The maximum loads of the TL system are also shown in Figure 8. The decrease in the maximum load was similar to that of the decrease in the adsorption coefficient. Both values began to decrease at 130 °C and saturated at temperatures above 150 °C. Furuta *et al.* [21] reported that the conformation of cellulose, hemicelluloses, and lignin are stabilized by heat treatment at temperatures over 100 °C. The ultrastructure densification in the cell wall results from the stabilization of wood substance. The energy of moisture adsorption may depend on the conformation of the wood substance, and the maximum load is related to the adhesive strength of the wood substance between cells. When the specimen was split under tensile stress in the tangential direction, the crack path typically followed interfaces between rays and tracheids, which are well known as the weakness planes. In spruce earlywood, both intercellular (separation of cells along the middle lamella) and transwall fracture occurred [16]. The compound middle lamella (CML) contains higher proportions of hemicelluloses than do secondary wall [22]. The heat treatment induces a conformation change of hemicelluloses caused by heating in CML more than in secondary walls. The change of CML may decrease the maximum load of the TL system.

#### 3.2.3. Adsorption Site Estimated by Hailwood–Horrobin Adsorption Equation

**Figure 9:**
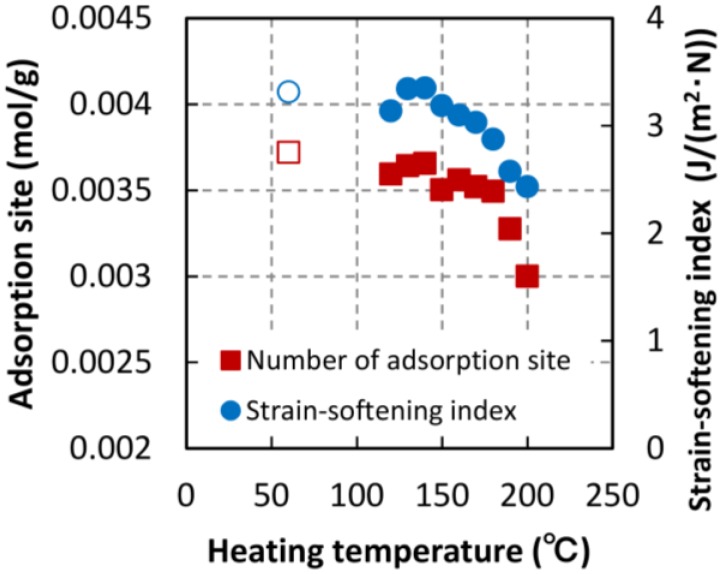
Number of adsorption site in heated specimens.

Figure 9 shows the change in the number of moisture adsorption sites with thermal treatment. These numbers were estimated by fitting the Hailwood-Horrobin adsorption equation to the moisture adsorption isotherms. The number of adsorption sites drastically decreased at temperature over 180 °C. The same behavior was observed in a previous report on the thermal treatment of poplar veneer [9]. According to specifications of the ThermoWood^®^ process, dried wood is heated to a temperature of at least 180 °C. With the degradation of hemicellulose, the concentration of water-absorbing hydroxyl groups decreases, and the dimensional stability of the ThermoWood^®^ improves compared to normal kiln–dried softwood [23]. When wood is heated to a temperature of more than 180 °C, moisture adsorption sites, namely, the water-absorbing hydroxyl groups of hemicellulose disappear abruptly.

The strain-softening index of TL systems was also shown in Figure 9. This decreasing trend in the number of adsorption sites is similar to the index trend. The strain-softening index may be related to the quantity of water-absorbing hydroxyl groups as well. The fracture process zone (FPZ) exits at the top of a crack [24,25]. Since fiber bridging is the primary toughening mechanism during crack propagation [26], we hypothesize that the hydrogen bonds of hydroxyl groups may influence the energy dissipated by the fiber bridging mechanism.

## 4. Conclusions

The cell wall structure of wood specimens heated to temperature between 120 and 200 °C for 1 h was examined by analyzing the fracture and moisture adsorption properties. Fracture properties were obtained using a single-edge notched bending test. We found that the fracture energy and fiber saturation point (estimated using the adsorption isotherm) began to decrease after thermal treatment above 150 °C. This indicates that the chemical structure of the cell wall is altered by heating above 150 °C, owing to the fact that the fracture energy depends on cell wall ultrastructure and the fiber saturation point is strongly influenced by high equilibrium moisture contents where the ultrastructure changes. The maximum load of heat-treated specimens began to decrease at temperatures above 130 °C, but saturated at temperatures above 150 °C. The Langmuir adsorption coefficient (related to the energy of adsorption) behaved similarly. The energy of adsorption may depend on the conformation of the wood substance. This indicates that the maximum load is influenced by the condition of the adsorption sites of the wood substance. Strain-softening index, which was obtained by dividing the fracture energy by the maximum load, abruptly decreased at 180 °C, a trend similar to that of the number of adsorption sites. It is known that hemicellulose pyrolytically decomposes at temperature above 180 °C, which indicates that the water-absorbing hydroxyl groups of hemicellulose may have an effect on the strain-softening behavior.
